# Supplementary material for: A Silicon Rhodamine‐fused Glibenclamide to Label and Detect Malaria‐infected Red Blood Cells
Source: Chembiochem. 2025 Mar 12;26(7):e202400628. doi: 10.1002/cbic.202400628 (PMC12002102; doi:10.1002/cbic.202400628)
Supplement: Supplementary file 1 — Supporting Information [file CBIC-26-e202400628-s001.pdf]

ChemBioChem

Supporting Information

A Silicon Rhodamine-fused Glibenclamide to Label and Detect Malaria-infected Red Blood Cells

Claudia Bastl, Cindy M. Close, Ingo Holtz, Blaise Gatin-Fraudet, Mareike Eis, Michelle Werum, Smilla Konrad, Laura Kneller, Kilian Roßmann, Christiane Huhn, Souvik Ghosh, Julia Ast, Dorien A. Roosen, Martin Lehmann, Volker Haucke, Luc Reymond, David J. Hodson, Philip Tinnefeld, Kai Johnsson, Viktorija Glembockyte,* Nicole Kilian,* and Johannes Broichhagen*

Supplemental Information for:

A silicon rhodamine-fused glibenclamide to label and detect malaria-infected red blood cells

Claudia Bastl[1*], Cindy M. Close[2*], Ingo Holtz[1*], Blaise Gatin-Fraudet[3], Mareike Eis[1], Michelle Werum[1], Smilla Konrad[1], Laura Kneller[3], Kilian Roßmann[3], Christiane Huhn[3], Souvik Ghosh[3], Julia Ast[X], Dorien A. Roosen[4], Martin Lehmann[3], Volker Haucke[4], Luc Reymond[5], David J. Hodson[X], Philip Tinnefeld[Y], Kai Johnsson[5,6], Viktorija Glembockyte[2†], Nicole Kilian[1,7†], Johannes Broichhagen[3,5,6†]

Affiliations

[1] Centre for Infectious Diseases, Parasitology Heidelberg University Hospital, Im Neuenheimer Feld 324, 69120 Heidelberg, Germany.

[2] Department of Chemistry and Center for NanoScience (CeNS), Ludwig-Maximilians-University, Butenandtstraße 5–13, 81377 Munich, Germany

[3] Leibniz-Forschungsinstitut für Molekulare Pharmakologie, Robert-Rössle-Straße 10, 13125 Berlin, Germany.

[4] Department of Molecular Pharmacology and Cell Biology, Leibniz-Forschungsinstitut für Molekulare Pharmakologie, Robert-Rössle-Straße 10, 13125 Berlin, Germany.

[5] Laboratory of Protein Engineering, Institut des Sciences et Ingénierie Chimiques, Sciences de Base, École Polytechnique Féderale Lausanne, Avenue Forel 2, 1015 Lausanne, Switzerland.

[6] Department of Chemical Biology, Max Planck Institute for Medical Research, Jahnstr. 29, 69120 Heidelberg, Germany.

[7] Department of Medical Biochemistry, Faculty of Basic Medical Sciences, Delta State University, Abraka, Nigeria

*Equal contribution

† Correspondence should be addressed to:

Viktorija.Glembockyte@cup.lmu.de

Broichhagen@fmp-berlin.de

Nicole.Kilian@med.uni-heidelberg.de; Nicole.Kilian@delsu.edu.ng

Table of Contents

1. General 3

2. Synthesis 4

2.1. 5-Chloro-2-methoxy-3-nitro-N-(4-sulfamoylphenethyl)benzamide (2) 4

2.2. 5-Chloro-N-(4-(N-(cyclohexylcarbamoyl)sulfamoyl)phenethyl)-2-methoxy-3-nitrobenzamide (3) 5

2.3. 3-Amino-5-chloro-N-(4-(N-(cyclohexylcarbamoyl)sulfamoyl)phenethyl)-2-methoxybenzamide (4) 6

2.4. 3-(6-Aminohexanamido)-5-chloro-N-(4-(N-(cyclohexylcarbamoyl)sulfamoyl)phenethyl)-2-methoxybenzamide (6) 7

2.5. 4-((6-((5-Chloro-3-((4-(N-(cyclohexylcarbamoyl)sulfamoyl)phenethyl)carbamoyl)-2-methoxyphenyl)amino)-6-oxohexyl)carbamoyl)-2-(7-(dimethylamino)-3-(dimethyliminio)-5,5-dimethyl-3,5-dihydrodibenzo[b,e]silin-10-yl)benzoate (SiR-glib) 8

3. NMR spectra 9

3.1. 5-Chloro-2-methoxy-3-nitro-N-(4-sulfamoylphenethyl)benzamide (2) 9

3.2. 5-Chloro-N-(4-(N-(cyclohexylcarbamoyl)sulfamoyl)phenethyl)-2-methoxy-3-nitrobenzamide (3) 10

3.3. 3-Amino-5-chloro-N-(4-(N-(cyclohexylcarbamoyl)sulfamoyl)phenethyl)-2-methoxybenzamide (4) 11

3.4. 3-(6-Aminohexanamido)-5-chloro-N-(4-(N-(cyclohexylcarbamoyl)sulfamoyl)phenethyl)-2-methoxybenzamide (6) 12

3.5. 4-((6-((5-Chloro-3-((4-(N-(cyclohexylcarbamoyl)sulfamoyl)phenethyl)carbamoyl)-2-methoxyphenyl)amino)-6-oxohexyl)carbamoyl)-2-(7-(dimethylamino)-3-(dimethyliminio)-5,5-dimethyl-3,5-dihydrodibenzo[b,e]silin-10-yl)benzoate (SiR-glib) 14

1. General

All chemical reagents and anhydrous solvents for synthesis were purchased from commercial suppliers (Sigma-Aldrich, Fluka, Acros, Fluorochem, TCI) and were used without further purification if not stated otherwise. Commercial coumarin 461 and methylene blue were HPLC purified before concentration assessment and measuring photophysical properties to ensure similar purity and composition as their synthesizes, deuterated counterparts. BG-TMR and BG-SiR were described before.[1]

NMR spectra were recorded at 300 K in deuterated solvents on a Bruker AVANCE III HD 600 equipped with a CryoProbe or on Bruker AV-III spectrometers using either a cryogenically cooled 5 mm TCI-triple resonance probe equipped with one-axis self-shielded gradients or room temperature 5 mm broadband probe and calibrated to residual solvent peaks ($^{1}H/^{13}C$ in ppm): DMSO-d_6 (2.50/39.52), MeOD-d_4 (3.31/49.00). Multiplicities are abbreviated as follows: s = singlet, d = doublet, t = triplet, q = quartet, p = pentet, h = heptet, br = broad, m = multiplet. Coupling constants J are reported in Hz. Spectra are reported based on appearance, not on theoretical multiplicities derived from structural information.

LC-MS was performed on an Agilent 1260 Infinity II LC System equipped with Agilent SB-C18 column (1.8 µm, 2.1 × 50 mm). Buffer A: 0.1% FA in H_2O Buffer B: 0.1% FA acetonitrile. The typical gradient was from 10% B for 0.5 min → gradient to 95% B over 5 min → 95% B for 0.5 min → gradient to 99% B over 1 min with 0.8 mL/min flow. Retention times (t_R) are given in minutes (min). Chromatograms were imported into Graphpad Prism8 and purity was determined by calculating AUC ratios.

Preparative or semi-preparative HPLC was performed on different instruments. An Agilent 1260 Infinity II LC System equipped with columns as followed: preparative column –Reprospher 100 C18 columns (10 µm: 50 x 30 mm at 20 mL/min flow rate; semi-preparative column – 5 µm: 250 x 10 mm at 4 mL/min flow rate. Eluents A (0.1% TFA in H2O) and B (0.1% TFA in MeCN) were applied as a linear gradient. Peak detection was performed at maximal absorbance wavelength. iii) A Waters e2695 system on a Supelco Ascentis® C18 HPLC Column (5 µm, 250 × 21.2 mm at 8 mL/min). Eluents A (0.1% TFA in H_2O) and B (0.1% TFA in MeCN) were applied as a linear gradient.

High resolution mass spectrometry was performed on an Agilent Technologies 6230 series accurate mass TOF LC-MS linked to an Agilent Technologies 1290 Infinity Series machine with a Thermo Accucore™ RP-MS column, 2.6 µm pore size, 30 × 2.1 mm, and a 3 min gradient from 5 to 99% aqueous MeCN with 0.1% TFA and MeCN with 0.1% TFA. flow rate: 0.8 mL/min; UV-detection: 220 nm, 254 nm, 300 nm.

2. Synthesis

2.1. 5-Chloro-2-methoxy-3-nitro-*N*-(4-sulfamoylphenethyl)benzamide (2)

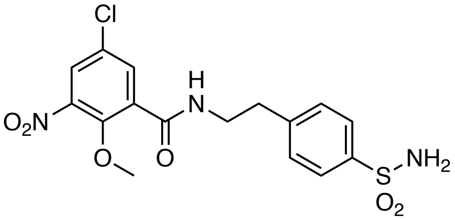

A round bottom flask was charged with 50 mg (0.21 mmol, 1.0 equiv.) 5-chloro-2-methoxy-3-nitrobenzoic acid (**1**) and dissolved in 1.2 mL DMSO and 97 µL (2.4 equiv.) DIPEA before 78.1 mg (0.25 mmol, 1.2 equiv.) of TSTU was added in one portion. The reaction mixture was stirred for 10 minutes before 51.8 mg (0.25 mmol, 1.2 equiv) of 4-(2-aminoethyl)benzenesulfonamide was added in one portion. The reaction mixture was stirred for additional 20 minutes before it was quenched with 10 mL of dH_2O, added dropwise under vigorous stirring. Finally, 1.2 mL of 1 M HCl was added to the reaction and the precipitate was collected by centrifugation at 4,000 rpm for 10 minutes. The supernatant was discarded, and the residue suspended in 10 mL dH_2O / 1.2 mL 1 M HCl, and sedimentation was performed again. After removing the supernatant, the residue was transferred to a round bottom flask with MeOH and all volatiles evaporated to dryness. 72 mg (0.17 mmol) of the desired product was obtained in 80% yield as a yellowish powder.

^{1}H NMR (300 MHz, DMSO-d_6): δ [ppm] = 8.71 (t, J = 5.5 Hz, 1H, NH), 8.16 (d, J = 2.8 Hz, 1H), 7.75 (d, J = 8.3 Hz, 2H), 7.73 (d, J = 2.7 Hz, 2H), 7.46 (d, J = 8.3 Hz, 2H), 7.32 (s, 2H, NH_2), 3.68 (s, 3H, CH_3), 3.51-3.59 (m, 2H), 2.92 (t, J = 7.1 Hz, 2H).

^{13}C NMR (75 MHz, DMSO-d_6): δ [ppm] = 163.5, 148.4, 144.5, 143.4, 142.2, 134.0, 132.9, 129.2, 127.5, 125.7, 125.4, 63.2, 34.4. (one carbon signal is missing as it's overlapping with DMSO signal)

HRMS (ESI): calc. for $C_{16}H_{17}ClN_3O_6S$ $[M+H]^+$: 414.0521, found: 414.0511.

2.2. 5-Chloro-*N*-(4-(*N*-(cyclohexylcarbamoyl)sulfamoyl)phenethyl)-2-methoxy-3-nitrobenzamide (3)

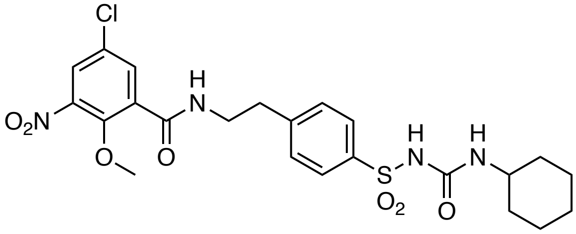

A round bottom flask was charged with 50 mg (120 µmol, 1.0 equiv.) of **2** and dissolved in 3 mL acetone before 33.2 mg (240 µmol, 2.0 equiv) of K_2CO_3 was added. The reaction mixture was heated to 70 °C and 23 µL (180 µmol, 22.6 mg, 1.5 equiv.) of cyclohexyl isocyanate diluted in 1 mL acetone was added dropwise. The reaction mixture was heated for an additional hour before 1 mL of 1 M HCl was added, and all volatiles were removed on a rotary evaporator. The residue was suspended in 10 mL dH_2O / 1 mL 1 M HCl and the precipitate was collected after centrifugation at 4,000 rpm for 10 minutes. After removing the supernatant, the residue was transferred to a round bottom flask with MeOH and all volatiles evaporated to dryness. 64 mg (128 µmol) of the desired product was obtained in quantitative yield as a white powder.

^{1}H NMR (600 MHz, DMSO-d_6): δ [ppm] = 10.29 (s, 1H, NH), 8.67 (t, J = 5.5 Hz, 1H, NH), 8.14 (d, J = 2.7 Hz, 1H), 7.82 (d, J = 8.3 Hz, 2H), 7.71 (d, J = 2.7 Hz, 1H), 7.50 (d, J = 8.3 Hz, 2H), 6.32 (d, J = 7.9 Hz, 1H, NH), 3.61 (s, 3H, CH_3), 3.57 (q, J = 6.5 Hz, 2H), 3.23-3.29 (m, 1H), 2.95 (t, J = 7.0 Hz, 2H), 1.61-1.67 (m, 2H), 1.55-1.61 (m, 2H), 1.46-1.51 (m, 1H), 1.17-1.25 (m, 2H), 1.05-1.15 (m, 3H).

^{13}C NMR (150 MHz, DMSO-d_6): δ [ppm] = 163.4, 150.35, 148.3, 145.0, 144.45, 138.2, 133.9, 132.85, 129.25, 127.4, 127.25, 125.3, 63.0, 48.0, 34.3, 32.2 (2C), 24.9, 24.1 (2C).

HRMS (ESI): calc. for $C_{23}H_{28}ClN_4O_7S$ $[M+H]^+$: 539.1362, found: 539.1392.

2.3. 3-Amino-5-chloro-*N*-(4-(*N*-(cyclohexylcarbamoyl)sulfamoyl)phenethyl)-2-methoxybenzamide (4)

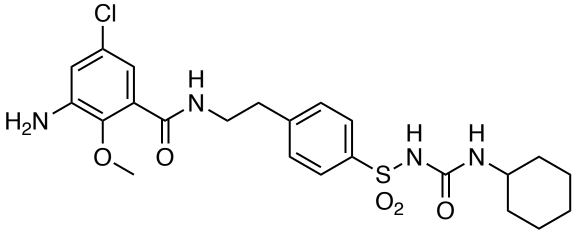

A round bottom flask was charged with 40 mg (75.3 µmol, 1.0 equiv.) of **3** under a nitrogen atmosphere and dissolved in 5 mL MeOH and 270 µL HOAc before 189 mg (12 mmol, 160 equiv.) of Zn dust was added in one portion. The reaction mixture was stirred for 1.5 hours at room temperature (solution turns red, and color fades over time) before it was centrifuged at 4,000 rpm for ten minutes to settle the remaining solids. The supernatant was collected and all volatiles were removed on a rotary evaporator. The crude was washed with 10 mL dH_2O, the solid filtered off and finally dried to obtain 34 mg (66 µmol) of the desired product in 92% yield as a white powder.

^{1}H NMR (600 MHz, MeOD-d_4): δ [ppm] = 7.91 (d, J = 8.2 Hz, 2H), 7.51 (d, J = 8.2 Hz, 2H), 6.86 (d, J = 2.6 Hz, 1H), 6.84 (d, J = 2.6 Hz, 1H), 3.69 (t, J = 7.0 Hz, 2H), 3.49 (s, 3H, CH_3), 3.05 (t, J = 7.0 Hz, 2H), 1.82-1.87 (m, 1H), 1.63-1.78 (m, 3H), 1.54-1.61 (m, 1H), 1.26-1.40 (m, 3H), 1.10-1.23 (m, 3H).

^{13}C NMR (150 MHz, MeOD-d_4): δ [ppm] = 168.0, 146.65, 144.7, 144.5, 140.1, 130.8, 130.6, 129.9, 128.8, 118.3, 117.8, 60.9, 50.1, 41.5, 35.9, 34.75, 33.8, 26.7, 26.5, 26.05, 25.8.

HRMS (ESI): calc. for $C_{23}H_{30}ClN_4O_5S$ $[M+H]^+$: 509.1620, found: 509.1625.

2.4. 3-(6-Aminohexanamido)-5-chloro-*N*-(4-(*N*-(cyclohexylcarbamoyl)sulfamoyl)phenethyl)-2-methoxybenzamide (6)

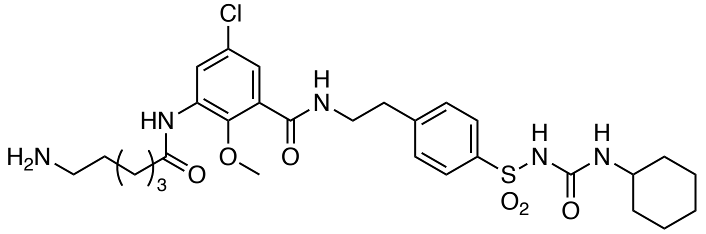

Under a nitrogen atmosphere, a round bottom flask was charged with 41 mg (117 µmol) of Fmoc-Ahx-OH and suspended in 1 mL $SOCl_2$. The reaction mixture was stirred for 1.5 hours at room temperature, and all volatiles were carefully removed under a stream of nitrogen in a well-ventilated fume hood. The residue was taken up in 1 mL of dry dioxanes, which was again carefully removed under a stream of nitrogen in a well-ventilated fume hood. Addition of 1 mL of dry dioxanes yielded a 117 mM stock solution, which was used immediately. A round bottom flask was charged with 10 mg (16.1 µmol, 1.0 equiv) of 4 dissolved in 2 mL of dry dioxanes and 10.8 µL of NEt_3, to which 420 µL of the acyl chloride (~3.0 equiv) was added dropwise under vigorous stirring. The reaction was stirred over night at room temperature before it was quenched with 1 mL of dH_2O and all volatiles were removed in vacuo. The crude material was taken up in 800 µL DMF and 40 µL piperidine and allowed to incubate for 1 hour at room temperature, before it was quenched with 50 µL HOAc and 200 µL dH_2O and subjected to RP-HPLC purification. The product containing fractions were pooled and 2.5 mg (3.4 µmol) of the desired product was obtained as a TFA salt after lyophilization in 21% yield over two steps as a white powder.

^{1}H NMR (600 MHz, DMSO-d_6): δ [ppm] = 10.34 (s, 1H, NH), 9.47 (s, 1H, NH), 8.43 (bs, 1H, NH), 8.16 (s, 1H), 7.83 (dd, J = 8.2 Hz, J = 1.8 Hz, 2H), 7.63 (bs, 2H, NH_2), 7.50 (dd, J = 8.2 Hz, J = 1.8 Hz, 2H), 7.12 (bs, 1H), 6.38 (d, J = 6.8 Hz, 1H, NH), 3.55 (bs, 5H, CH_2 and CH_3), 3.23-3.31 (m, 1H), 2.95 (bt, J = 6.8 Hz, 2H), 2.75-2.81 (m, 2H), 2.45 (bt, J = 6.8 Hz, 2H), 1.51-1.67 (m, 8H), 1.44-1.51 (m, 1H), 1.30-1.37 (m, 2H), 1.16-1.26 (m, 2H), 1.07-1.15 (m, 3H).

^{19}F NMR (564 MHz, DMSO-d_6): δ [ppm] = -73.8.

^{13}C NMR (150 MHz, DMSO-d_6): δ [ppm] = 172.0, 164.6, 150.4, 146.4, 145.1, 138.2, 133.2, 131.2, 129.2, 127.2, 127.0, 123.0, 122.9, 61.4, 48.0, 40.0, 38.7, 35.7, 34.4, 32.2 (2C), 26.8, 25.4, 24.9, 24.5, 24.1 (2C).

HRMS (ESI): calc. for $C_{23}H_{27}ClN_4O_7S$ $[M+H]^+$: 622.2461, found: 622.2438.

2.5. 4-((6-((5-Chloro-3-((4-(*N*-(cyclohexylcarbamoyl)sulfamoyl)phenethyl)carbamoyl)-2-methoxyphenyl)amino)-6-oxohexyl)carbamoyl)-2-(7-(dimethylamino)-3-(dimethyliminio)-5,5-dimethyl-3,5-dihydrodibenzo[*b,e*]silin-10-yl)benzoate (SiR-glib)

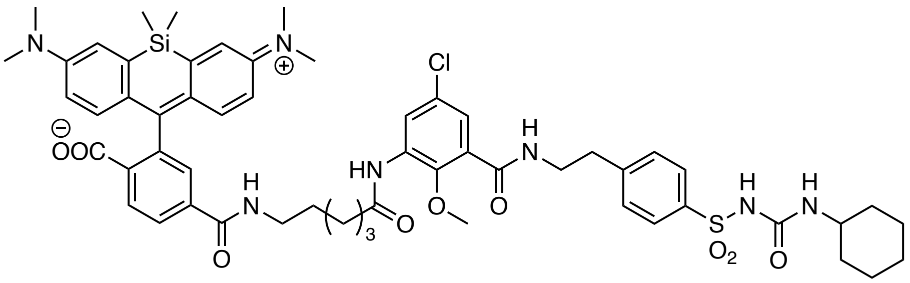

An Eppendorf tube was charged with 1.8 mg (3.9 µmol, 1.2 equiv.) of SiR-6-COOH and dissolved in 0.5 mL DMSO and 2.0 µL DIPEA before 1.4 mg (4.5 µmol, 1.5 equiv.) of TSTU was added in one portion. The reaction mixture was allowed to incubate for 10 minutes before it was added to an Eppendorf tube containing 2.0 mg (3.2 µmol, 1.0 equiv.) of 6 dissolved in 0.5 mL DMSO. The reaction mixture was vortexed and allowed to incubate for 2 hours, before it was quenched with 20 µL HOAc and 100 µL dH2O and subjected to RP-HPLC purification. The product containing fractions were pooled and 1.7 mg (1.6 µmol) of the desired product was obtained after lyophilization in 49% yield over two steps as a blue powder.

^{1}H NMR (600 MHz, DMSO-d$_6$): δ [ppm] = 10.29 (s, 1H, NH), 9.44 (s, 1H, NH), 8.43 (t, J = 5.5 Hz, NH), 8.30 (d, J = 8.5 Hz, 1H), 8.21 (d, J = 8.5 Hz, 1H), 8.16 (bd, J = 2.4 Hz, 1H), 7.88 (t, J = 5.5 Hz, NH), 7.82 (d, J = 8.3 Hz, 2H), 7.77 (bs, 1H), 7.50 (d, J = 8.3 Hz, 2H), 7.12 (d, J = 2.7 Hz, 1H), 7.04 (bs, 2H), 6.69 (bs, 4H), 6.32 (d, J = 7.9 Hz, 1H, NH), 3.55 (m, 4H), 3.23-3.27 (m, 1H), 3.02-3.05 (m, 1H), 2.94 (bs, 12H), 2.87 (bs, 3H), 2.75 (bs, 1H), 2.43 (t, J = 7.7 Hz, 1H), 2.29 (t, J = 7.4 Hz, 1H), 1.95-2.02 (m, 1H), 1.62-1.67 (m, 1H), 1.56-1.59 (m, 2H), 1.40-1.49 (m, 2H), 1.19-1.32 (m, 4H), 1.08-1.14 (m, 1H), 0.94 (d, J = 6.6 Hz, 1H), 0.85 (t, J = 7.0 Hz, 1H), 0.62 (s, 3H), 0.53 (s, 3H).

^{19}F NMR (564 MHz, DMSO-d$_6$): δ [ppm] = -73.8.

HRMS (ESI): calc. for $C_{56}H_{66}ClN_7O_9SSi$ [M+H]$^+$: 1076.4173, found: 1076.4180.

3. NMR spectra

3.1. 5-Chloro-2-methoxy-3-nitro-*N*-(4-sulfamoylphenethyl)benzamide (2)

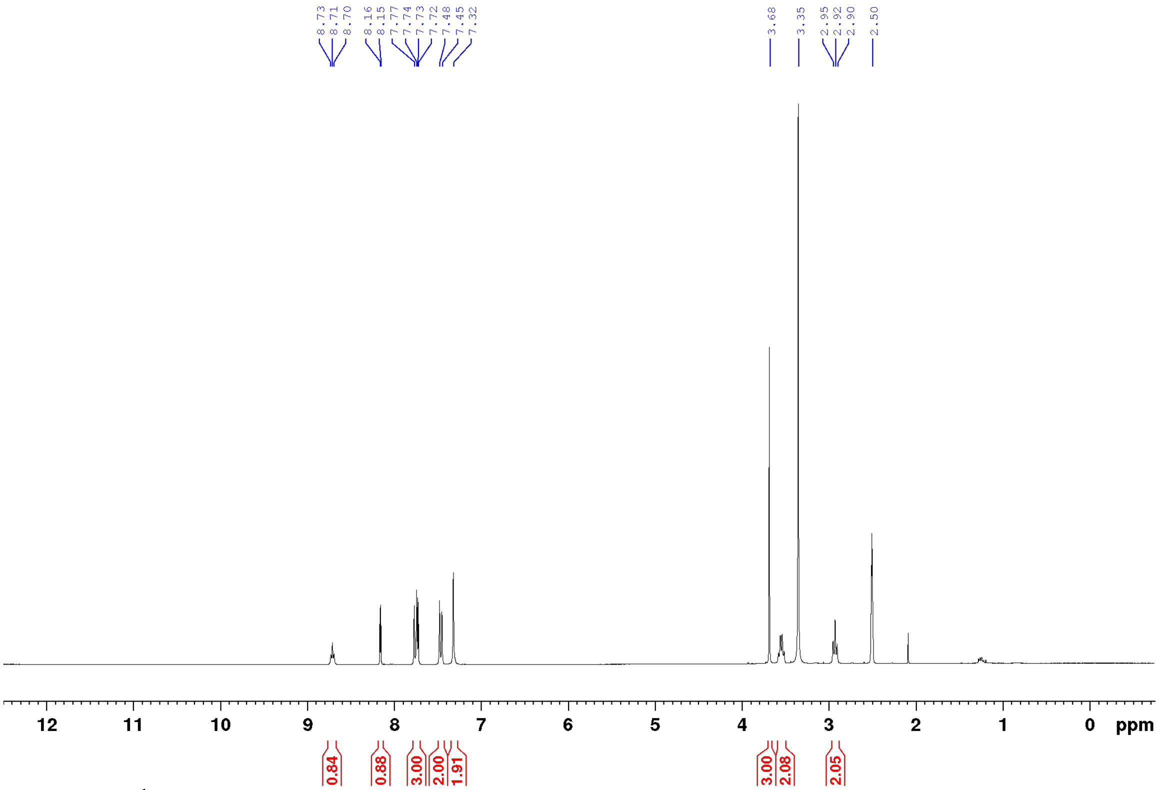

Figure S1. ^{1}H NMR spectra of compound **2** in DMSO-d$_6$.

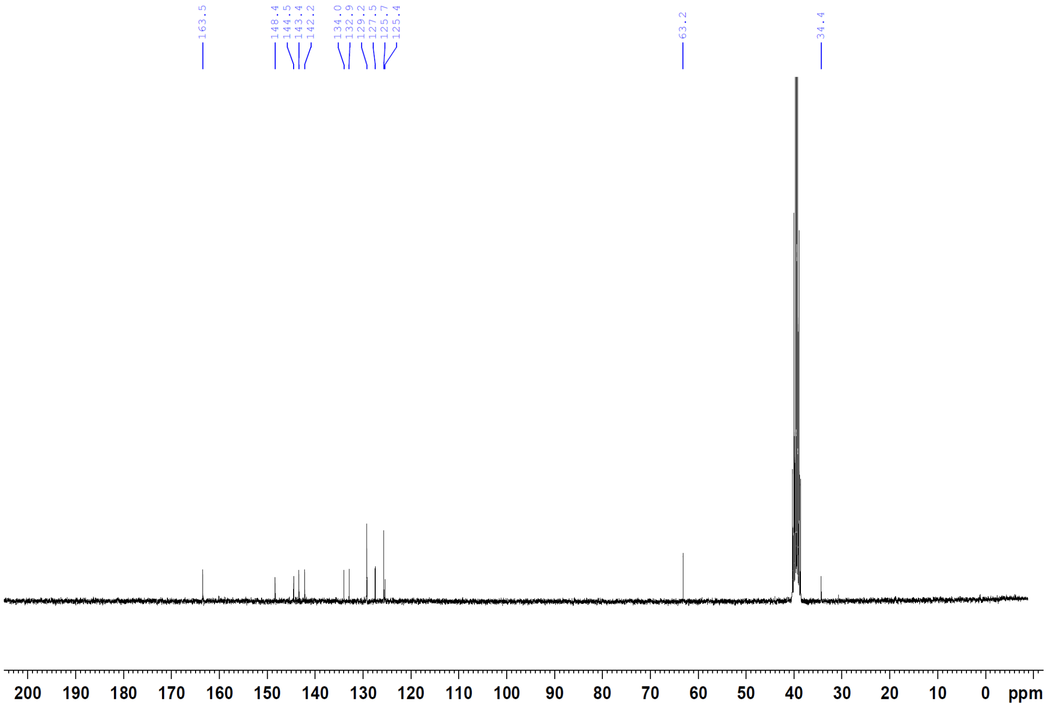

Figure S2. ^{13}C NMR spectra of compound **2** in DMSO-d$_6$.

3.2. 5-Chloro-*N*-(4-(*N*-(cyclohexylcarbamoyl)sulfamoyl)phenethyl)-2-methoxy-3-nitrobenzamide (3)

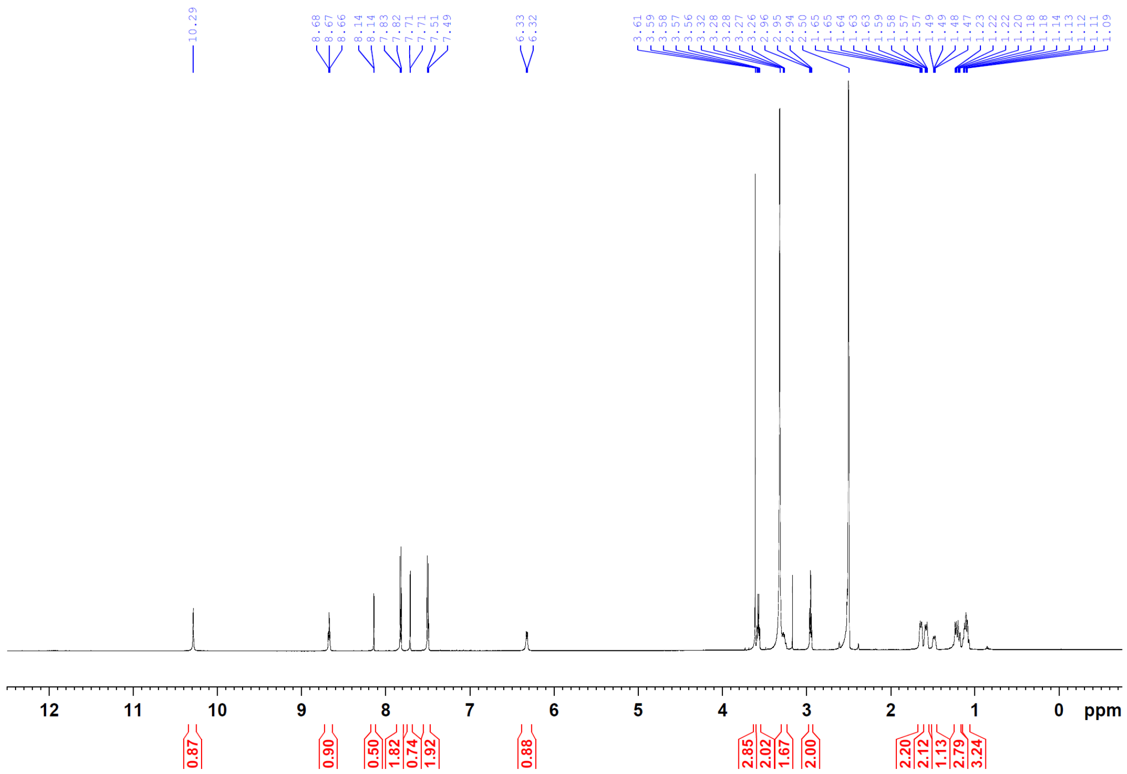

Figure S3. ^{1}H NMR spectra of compound **3** in DMSO-d$_6$.

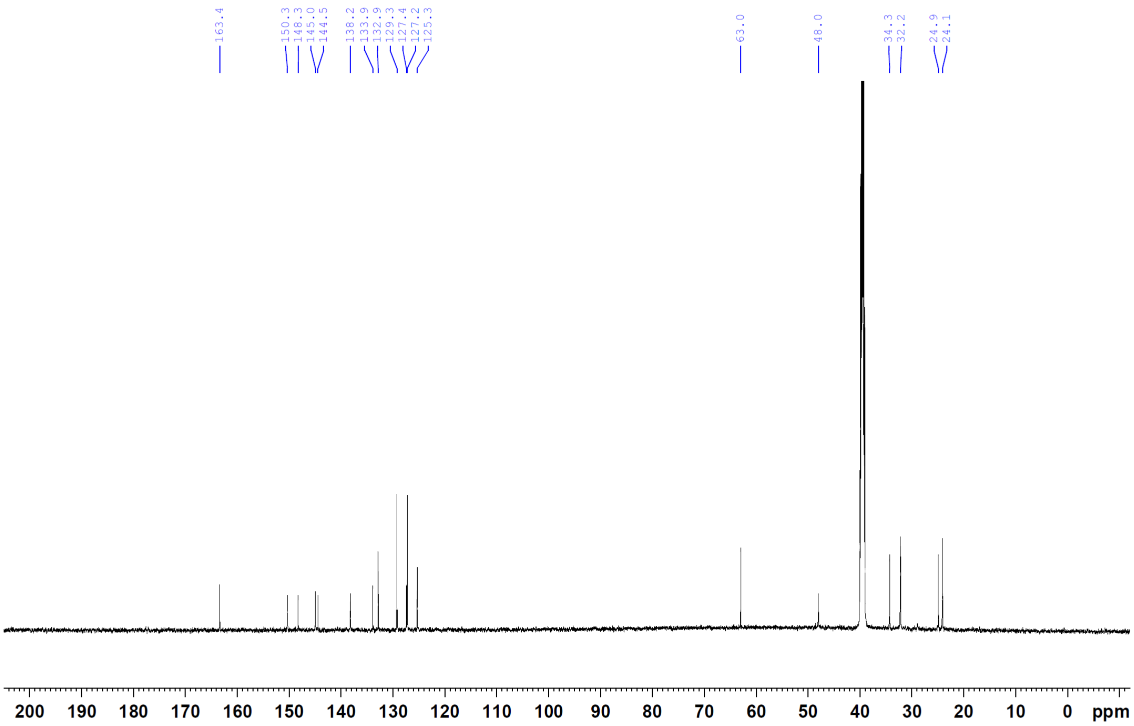

Figure S4. ^{13}C NMR spectra of compound **3** in DMSO-d$_6$.

3.3. 3-Amino-5-chloro-*N*-(4-(*N*-(cyclohexylcarbamoyl)sulfamoyl)phenethyl)-2-methoxybenzamide (4)

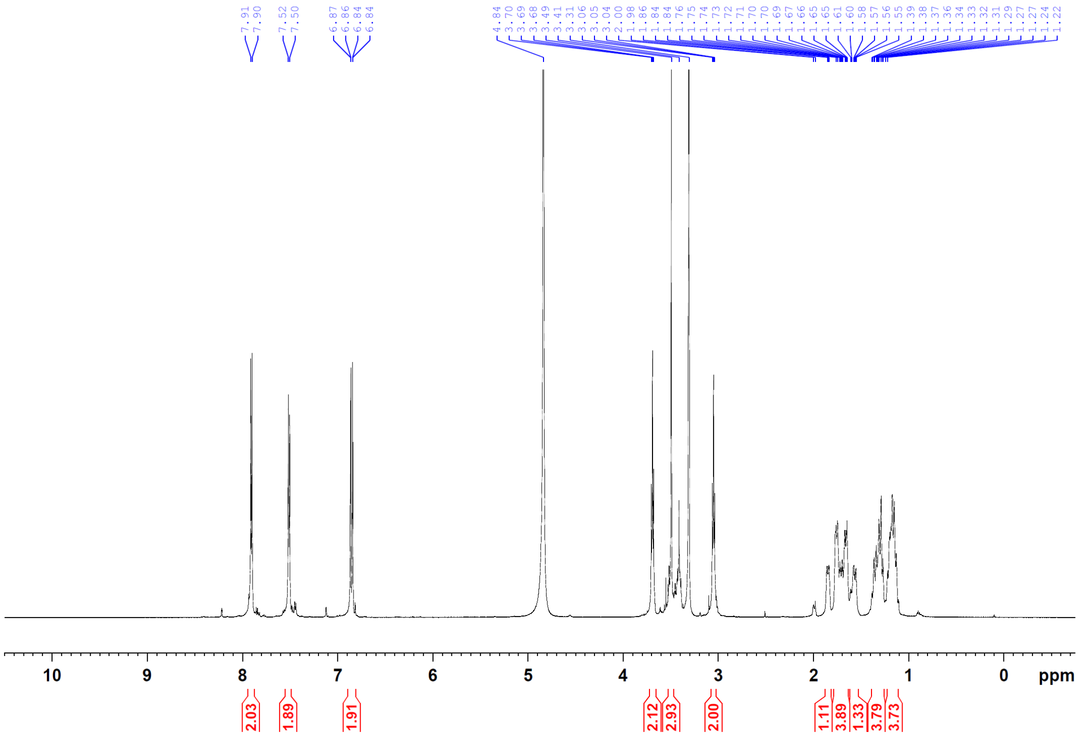

Figure S5. ^{1}H NMR spectra of compound **4** in MeOD-d_4.

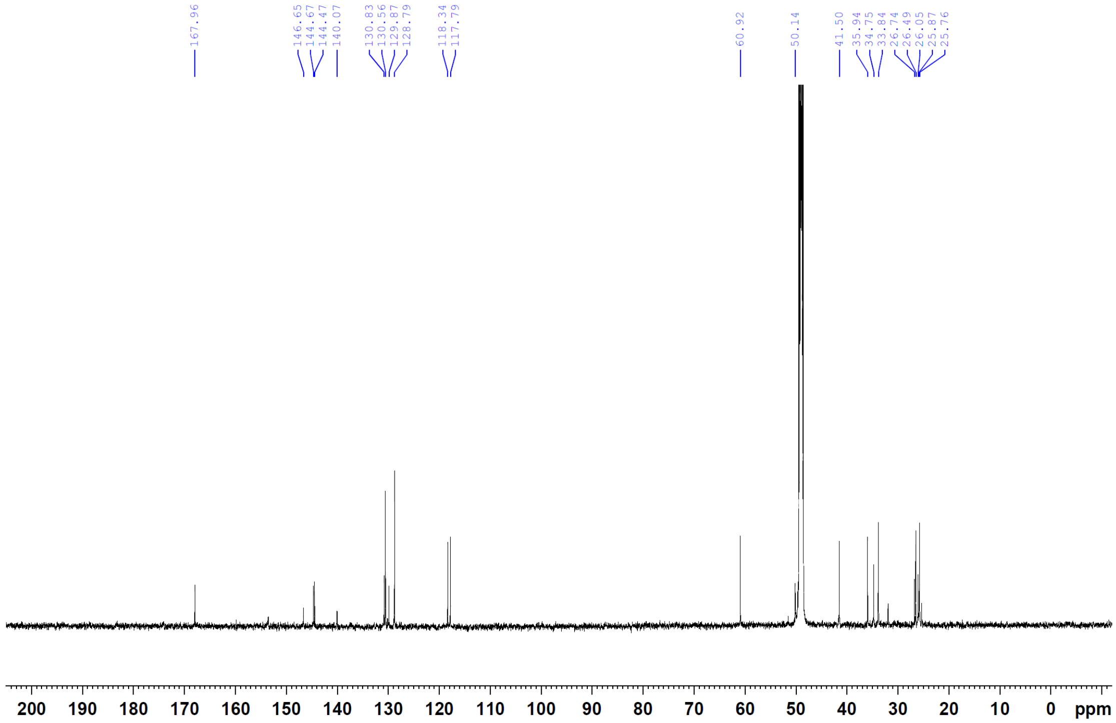

Figure S6. ^{13}C NMR spectra of compound **4** in MeOD-d_4.

3.4. 3-(6-Aminohexanamido)-5-chloro-*N*-(4-(*N*-(cyclohexylcarbamoyl)sulfamoyl)phenethyl)-2-methoxybenzamide (6)

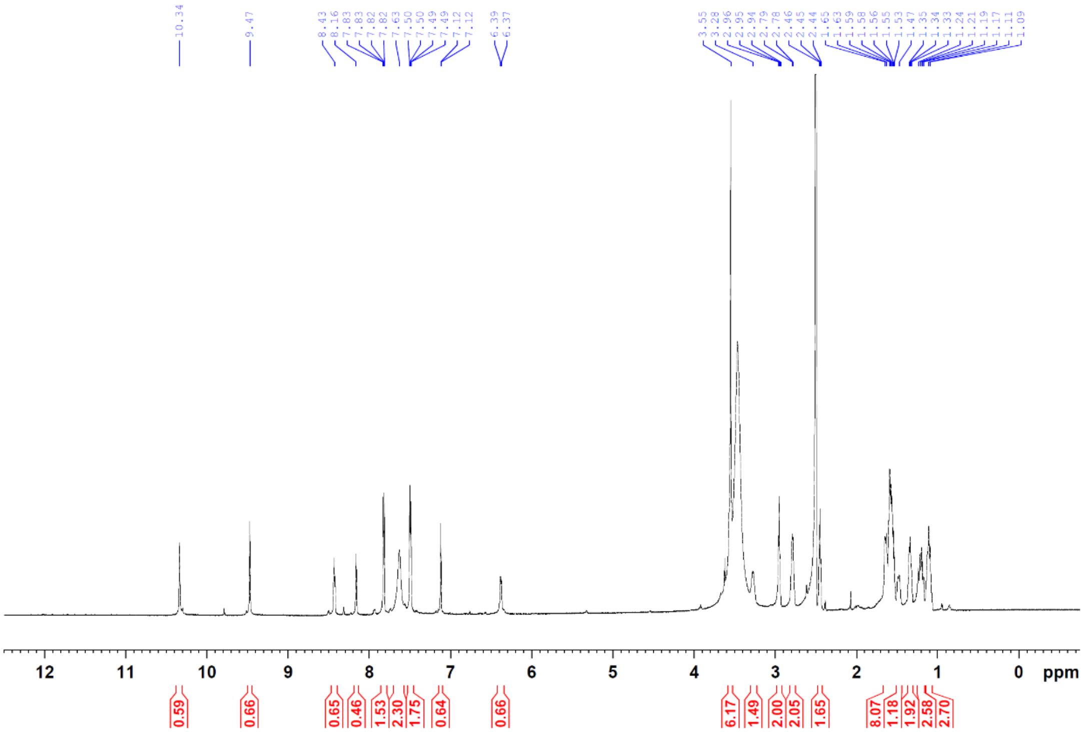

Figure S7. ^{1}H NMR spectra of compound **6** in DMSO-d$_6$.

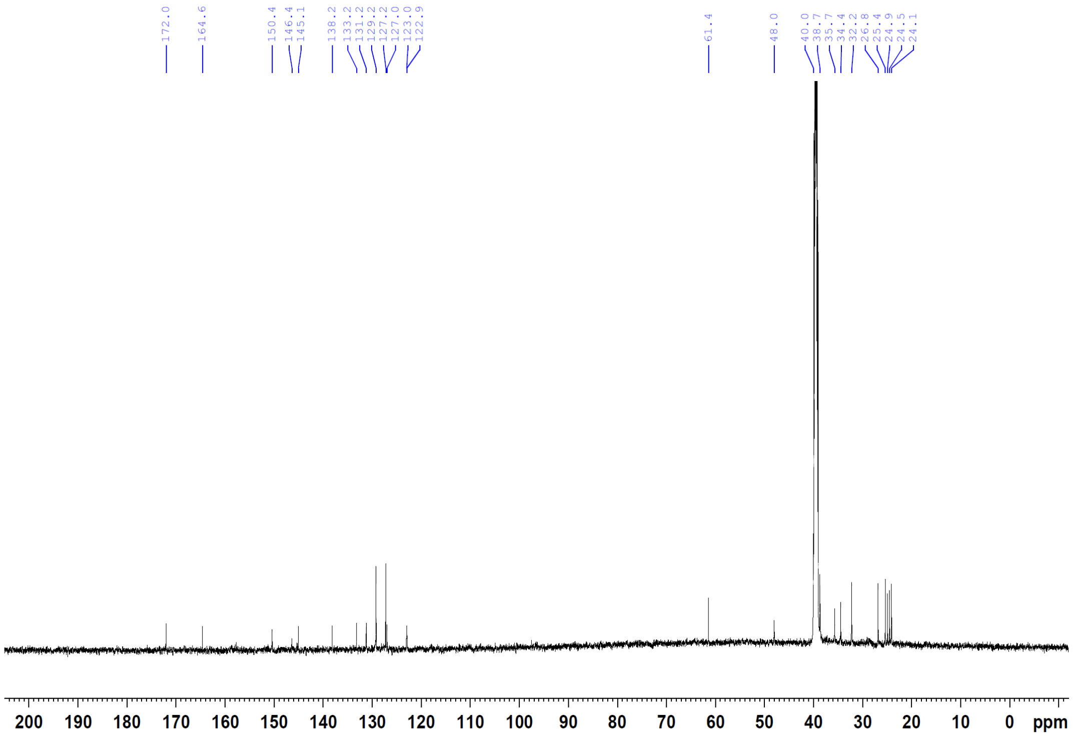

Figure S8. ^{13}C NMR spectra of compound **6** in DMSO-d$_6$.

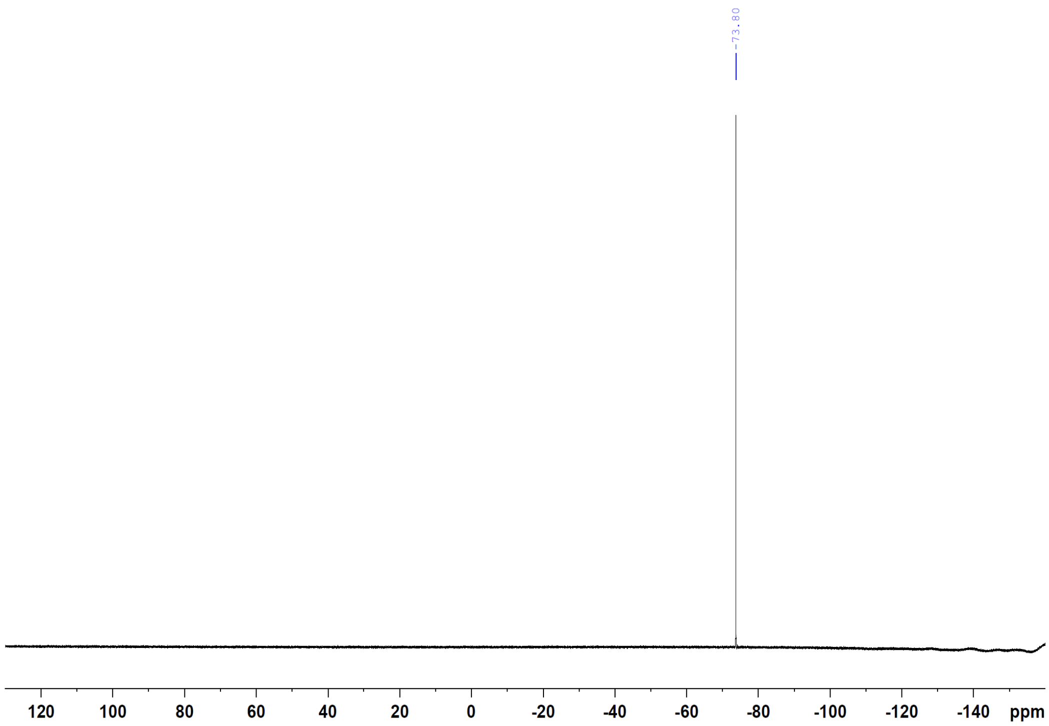

Figure S9. ^{19}F NMR spectra of compound **6** in DMSO-d_6.

3.5. 4-((6-((5-Chloro-3-((4-(*N*-(cyclohexylcarbamoyl)sulfamoyl)phenethyl)carbamoyl)-2-methoxyphenyl)amino)-6-oxohexyl)carbamoyl)-2-(7-(dimethylamino)-3-(dimethyliminio)-5,5-dimethyl-3,5-dihydrodibenzo[*b*,*e*]silin-10-yl)benzoate (SiR-glib)

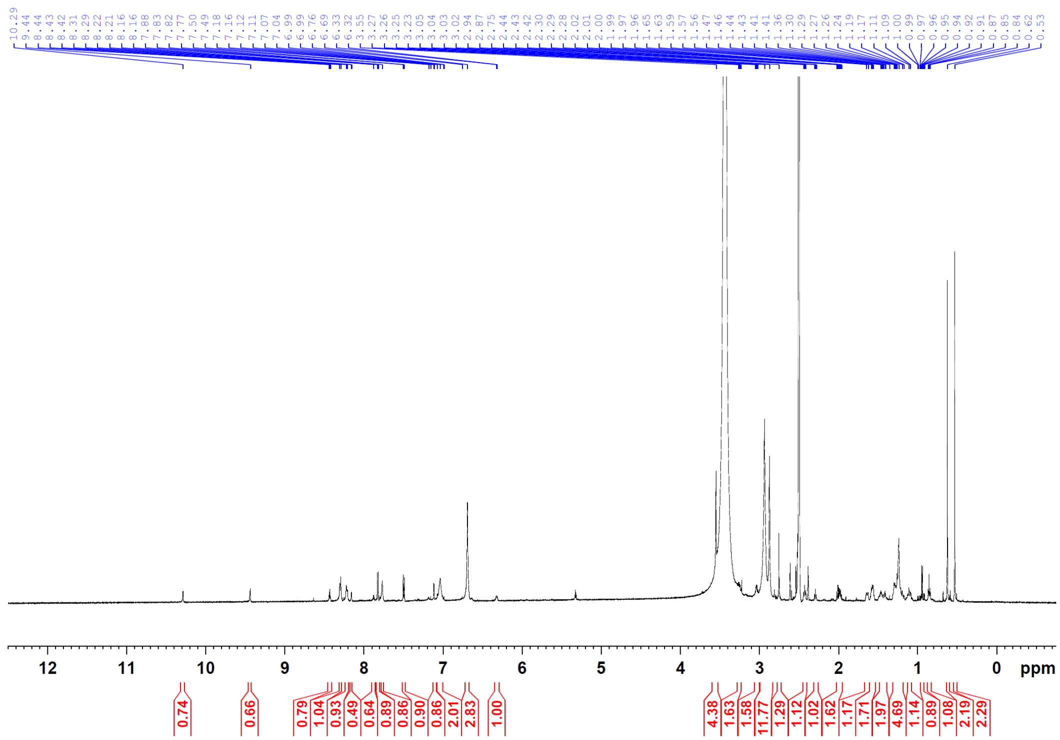

Figure S10. ^{1}H NMR spectra of compound **SiR-glib** in DMSO-d$_6$.

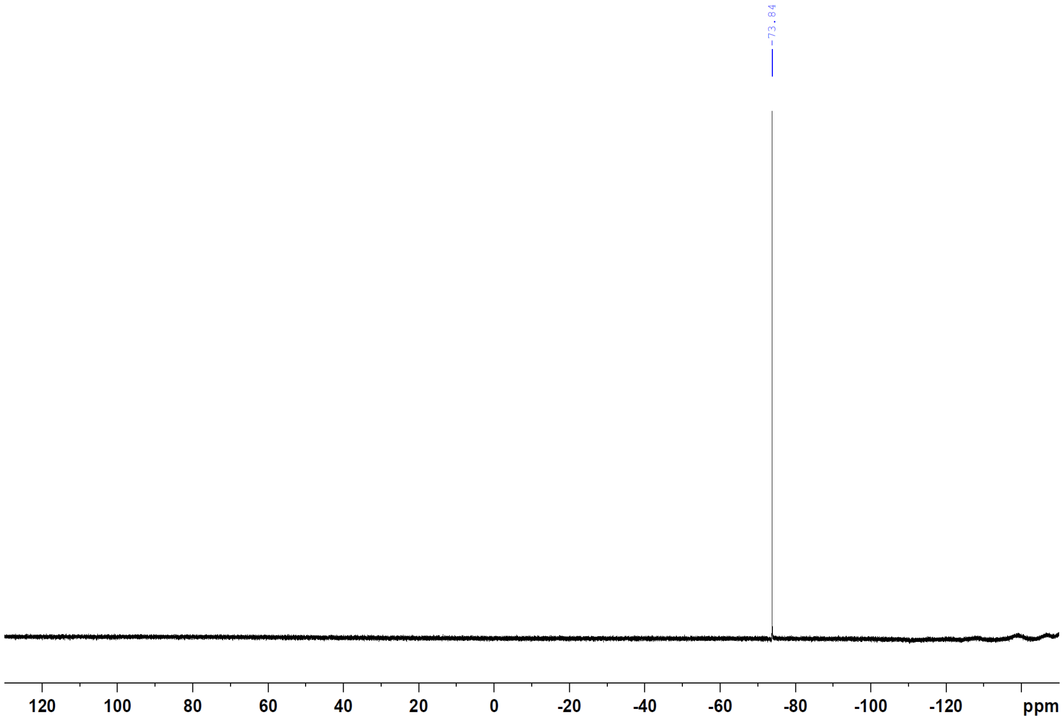

Figure S11. ^{19}F NMR spectra of compound **SiR-glib** in DMSO-d$_6$.

4. Supplementary Figures

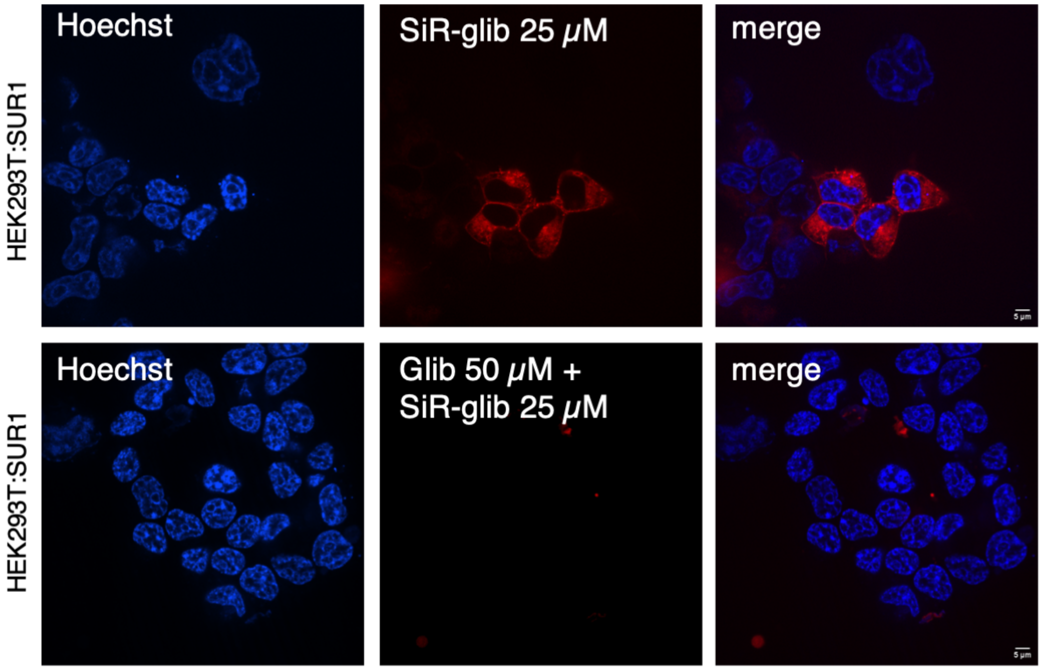

Figure S12. SUR1 transfected HEK293 cells. Scale bar = 5 um.

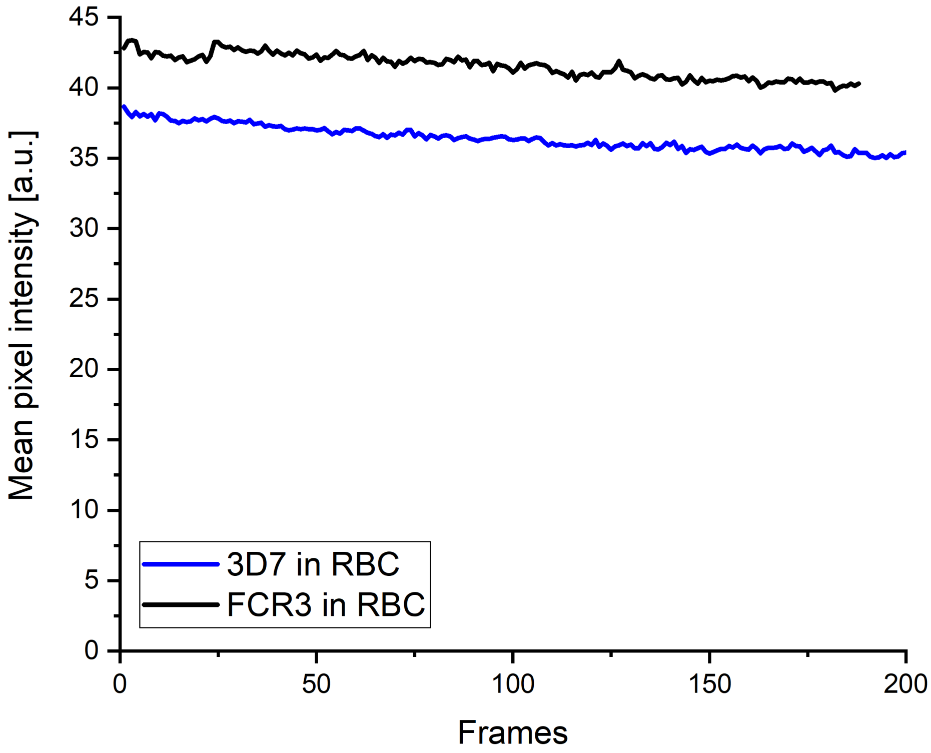

Figure S13. Photostability of SiR-glib in infected RBCs.

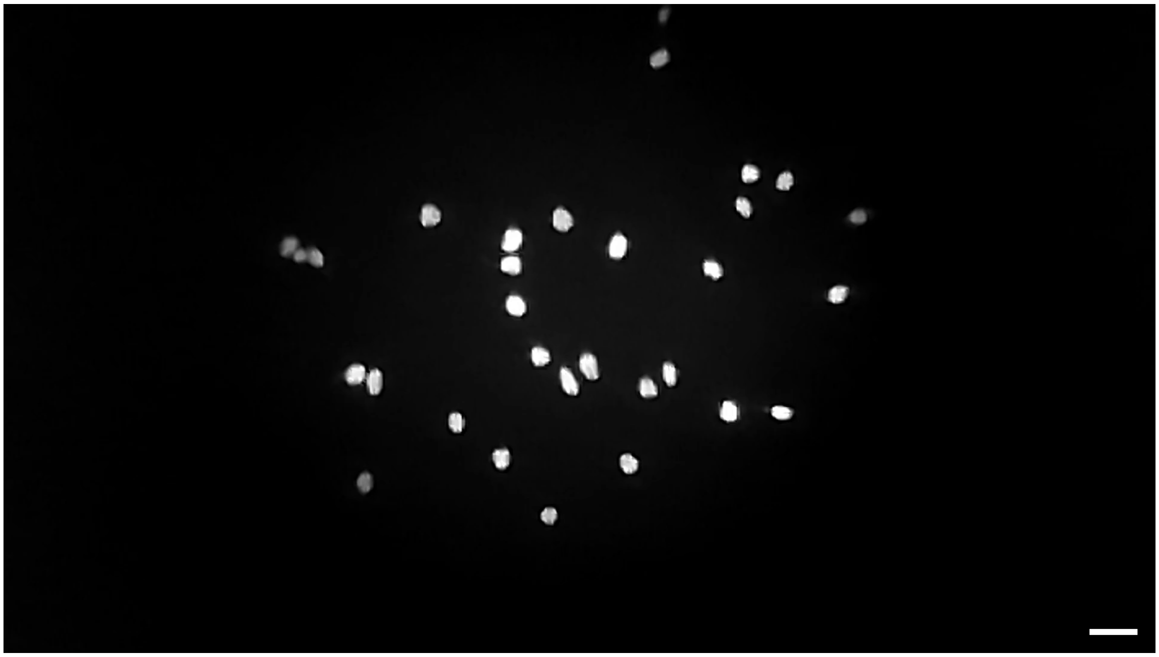
Figure S14. HeLa cells stained with SiR-DNA and imaged on the portable microscope.
